# Over-Expression of DSCAM and COL6A2 Cooperatively Generates Congenital Heart Defects

**DOI:** 10.1371/journal.pgen.1002344

**Published:** 2011-11-03

**Authors:** Tamar R. Grossman, Amir Gamliel, Robert J. Wessells, Ouarda Taghli-Lamallem, Kristen Jepsen, Karen Ocorr, Julie R. Korenberg, Kirk L. Peterson, Michael G. Rosenfeld, Rolf Bodmer, Ethan Bier

**Affiliations:** 1 Section of Cell and Developmental Biology, University of California San Diego, La Jolla, California, United States of America; 2 Howard Hughes Medical Institute, School of Medicine, University of California San Diego, La Jolla, California, United States of America; 3 University of Michigan, Ann Arbor, Michigan, United States of America; 4 Sanford-Burnham Medical Research Institute, La Jolla, California, United States of America; 5 The Brain Institute, School of Medicine, University of Utah, Salt Lake City, Utah, United States of America; 6 School of Medicine, University of California San Diego, La Jolla, California, United States of America; Stanford University School of Medicine, United States of America

## Abstract

A significant current challenge in human genetics is the identification of interacting genetic loci mediating complex polygenic disorders. One of the best characterized polygenic diseases is Down syndrome (DS), which results from an extra copy of part or all of chromosome 21. A short interval near the distal tip of chromosome 21 contributes to congenital heart defects (CHD), and a variety of indirect genetic evidence suggests that multiple candidate genes in this region may contribute to this phenotype. We devised a tiered genetic approach to identify interacting CHD candidate genes. We first used the well vetted *Drosophila* heart as an assay to identify interacting CHD candidate genes by expressing them alone and in all possible pairwise combinations and testing for effects on rhythmicity or heart failure following stress. This comprehensive analysis identified DSCAM and COL6A2 as the most strongly interacting pair of genes. We then over-expressed these two genes alone or in combination in the mouse heart. While over-expression of either gene alone did not affect viability and had little or no effect on heart physiology or morphology, co-expression of the two genes resulted in ≈50% mortality and severe physiological and morphological defects, including atrial septal defects and cardiac hypertrophy. Cooperative interactions between DSCAM and COL6A2 were also observed in the H9C2 cardiac cell line and transcriptional analysis of this interaction points to genes involved in adhesion and cardiac hypertrophy. Our success in defining a cooperative interaction between DSCAM and COL6A2 suggests that the multi-tiered genetic approach we have taken involving human mapping data, comprehensive combinatorial screening in *Drosophila*, and validation *in vivo* in mice and in mammalian cells lines should be applicable to identifying specific loci mediating a broad variety of other polygenic disorders.

## Author Summary

A large fraction of human genes may contribute to polygenic disorders, yet few experimental methods for identifying such genes are currently available. For example, with regard to congenital heart defects (CHD) caused by extra copies of genes on chromosome 21 in Down syndrome patients, it is not known which genes contribute to this complex phenotype. In this paper, we identify two genes, DSCAM and COL6A2 that interact strongly to produce CHD when over-expressed at modest levels in the mouse heart. These two genes were identified as the most strongly interacting pair of CHD candidate genes when over-expressed in the *Drosophila* heart, where they disrupted several indices of heart function. We then over-expressed these genes in the mouse heart alone or in combination and found that while expression of either gene alone had little or no effect, co-expression of the genes, as in flies, lead to severe cooperative defects in heart physiology and morphology. The strategy we have followed in this study is broadly applicable to identifying genes involved in other polygenic disorders, such as obesity, autism, and schizophrenia, which have been linked to altered copy number of multiple genes.

## Introduction

The Online Inheritance in Man (OMIM) lists over 3500 loci which when mutated give rise to heritable human disease. Approximately one third of these disorders are dominant, most of these cases being due to dosage sensitive requirements for gene function (i.e., haploinsufficiency or elevated gene activity) and a minority resulting from the production of an aberrant interfering protein product as has been extensively studied for peripheral neuropathies [1], [2]. In addition to single locus disorders resulting from altered gene dose, a large fraction of the genome may be involved in multi-locus complex disorders resulting from alterations in gene dose due to heterozygosity for macroscopic deletions or duplications, smaller chromosomal lesions resulting in copy number variation (CNV) [3], [4], or interactions between two or more genes in separate genomic intervals (e.g., as identified by the HapMap initiative [5]).

Given the large numbers of potentially interacting loci that could underlie polygenic disorders, systematic approaches to identify such loci are urgently needed. In the current study, we present a multi-tiered genetic approach that could be generalized to a broader range of disorders, to identify genes that interact to cause congenital heart defects (CHD). Based on human genetic data delimiting a short interval on the distal tip of chromosome 21 containing a small set of candidate genes which may contribute to CHD in human DS patients [6]–[10], we first employed *Drosophila* as a model to systematically examine the effect of over-expressing these candidate genes individually or in pairwise combinations in the pumping heart tube of adult flies as well as in a neurologically relevant tissue (the eye). Although the fly heart is a much less complex structure than its vertebrate counterparts, it has several important basic properties common to all hearts including developmental genes involved in specifying the heart primordium [11], proteins mediating periodic contractility, ion channels responsible for rhythmic beating, and morphological adaptations required for directional fluid pumping (e.g., valves) [12], as well as manifesting age-dependent deterioration [13]. In addition to being able to test many genetic combinations rapidly and to target gene over-expression to specific cell types such as the heart or eye, the fly provides a relatively stringent system for identifying genetic interactions since dominant phenotypes resulting from altering the dose of single genes are far less common than in humans.

Our comprehensive screening of CHD candidate genes in *Drosophila* identified DSCAM and COL6A2 as the most strongly interacting pair of genes. The effect of modestly over-expressing these genes in the mouse heart was then examined and, as in flies, these two genes interacted synergistically to cause defects in heart morphology and physiology. Over-expression of DSCAM and COL6A2 also resulted in a transcriptional response in cardiac H9C2 cells. Consistent with the known cell biological roles of these two genes in mediating cell-matrix adhesion, we found clear transcriptional signals for genes involved in cell adhesion as well as genes involved in cardiac disease. We discuss the prospects of using similar multi-tiered genetic approaches to identifying genes involved in other polygenic disorders.

## Results

### DSCAM and COL6A2 cooperatively disrupt heart function in flies

Congenital heart defects are observed in approximately 50% of DS patients and the genes responsible for this phenotype have been mapped to a small candidate region near the tip of chromosome 21 [9], [10]. Several of the genes included in this interval are known to be expressed in the heart and among this group a subset encode extracellular proteins or proteins interacting with them: SH3BGR, DSCAM, COL6A1, COL6A2 and COL18. Homologs of all human DS CHD candidate genes that were chosen for study are present in *Drosophila* (Table S1).

**Figure 1:**
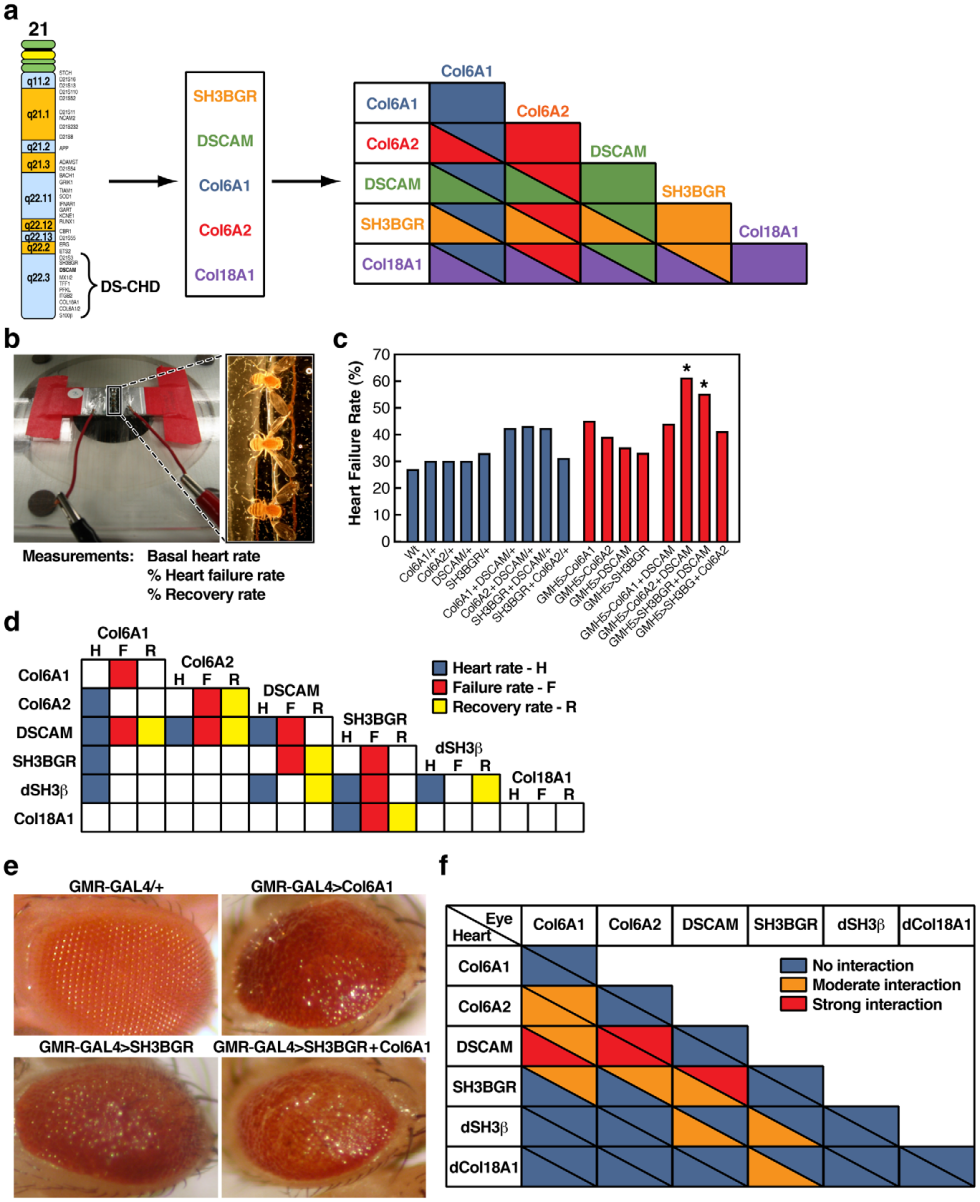
Survey of heart defects caused by over-expression of CHD candidate genes in the fly. a) Scheme depicting how candidate CHD genes from the distal region of chromosome 21 were screened by expressing them in the fly heart individually and in all possible pair-wise combinations. b) Electrical pacing heart performance stress chamber (left). Enlarged view of flies in pacing chamber (right). c) Example of increased heart failure rate in flies mis-expressing particular combinations of CHD candidate genes in the heart. The heart failure phenotype is presented as the percentage of flies whose heart fibrillated or stopped immediately following the pacing regimen (N = 200). (*Chi square p<0.05 for DSCAM+ COL6A2 and DSCAM+ SH3BGR). d) Interaction grid summarizing interactions between CHD candidate genes in the fly heart resulting in particular cardiac phenotypes. H  =  Heart rate, F  =  failure rate (% of flies that exhibited heart asystole or fibrillation immediately following the electrical pacing regime) and R  =  recovery rate (% of flies that exhibited recovered heart rate 2 minutes after the end of electrical pacing). Colored backgrounds indicate significant difference in heart function relative to non-expressing UAS controls (Chi square, p<0.05). Blue  =  heart rate; Red  =  stress-induced failure rate; Yellow  =  recovery rate following heart failure. e) Example of a genetic interaction between the CHD candidate genes SH3BGR and COL6A1 in the fly eye using the GMR-GAL4 driver. f) Summary of all genetic interactions between CHD candidate genes in the fly heart (lower triangles in each interaction box) and eye (upper triangles in each interaction box). Blue  =  no detectable interaction, orange  =  moderate interaction and red  =  strong interaction. The combination exhibiting the strongest interactions in both the heart and eye was DSCAM plus COL6A2.

We assayed the effect of over-expressing mammalian candidate CHD genes and their *Drosophila* orthologs selectively in the fly heart using the UAS/GAL4 trans-activation system [14]. Several independent UAS transgenic lines were generated for each of the mammalian and fly candidate genes, and these genes were expressed individually and in all pair-wise combinations in the *Drosophila* myocardium using the heart-specific GMH5-GAL4 driver [15] (Figure 1a). We assayed the effects of over-expressing CHD genes by measuring basal heart rate and by testing for heart failure following stress [15]. For the stress test, adult flies were subjected to a heart-pacing paradigm in which the heart rate was doubled (i.e., electrically stimulated to 6 Hz) for a period of 30 seconds. Following pacing, we monitored the proportion of flies with consequent cardiac dysfunction (termed here ‘heart failure’) and their recovery after 2 minutes [15] (Figure 1b, 1c and Table S2). In these performance tests, heart dysfunction was manifested by uncoordinated fibrillation or protracted periods of non-beating (asystole).

When expressed individually in the fly heart, several genes caused an increase in stress-induced cardiac dysfunction compared to controls for at least one of the three parameters tested, and three genes (DSCAM, COL6A2, and SH3BGR) altered two indices of heart function (Figure 1d and Table S2). Using these effects as a baseline, we next tested for cooperative interactions among CHD candidate genes by co-expressing them in all possible pair-wise combinations. The criterion we used to define a genetic interaction between two candidate genes was that the effect of co-expressing single copies of two genes was significantly greater than that caused by expressing two copies of each gene separately. This analysis revealed that the strongest interacting gene combinations were DSCAM+COL6A2 and DSCAM+SH3BGR (Figure 1c, 1d and Table S2). For these two genetic combinations, all three indices of heart function, heart rate, failure, and recovery rate, were altered (p<0.05). As an example of a cooperative effect, expression of either DSCAM or COL6A2 alone resulted in ≈35% heart failure rate (N = 200 for each genotype), but when these two genes were co-expressed, the failure rate nearly doubled to 60% (N = 200; p<0.05) (Figure 1c). Similarly, co-expression of DSCAM+COL6A1 or SH3BGR+dCOL18A1 resulted in significant perturbation of all 3 parameters tested (Figure 1d). Notably, three of the interacting genes (DSCAM, COL6A2, and SH3BGR) also had moderate effects when expressed individually. This comprehensive combinatorial study of CHD candidate genes in the fly heart revealed that over-expression of DSCAM caused the greatest disruption of heart function and that co-expression with COL6A2 most effectively potentiated this effect on all heart parameters scored.

In parallel to testing for defects in heart performance, we examined the effect of expressing CHD candidate genes in the fly eye, which is another widely used assay system for genetic interactions and a well established model for defining mechanisms underlying neurological disorders. As in the case of the heart, we expressed each CHD candidate gene alone and in all pair wise combinations using the eye specific GMR-GAL4 driver and observed varying degrees of roughened eyes (Figure 1e, 1f) ranging from mild to moderately disorganized eyes. As in the case of the heart expression experiments, we used these single gene phenotypes to assess potential cooperative effects of co-expressing candidate genes in all pairwise combinations. Again, we found several instances in which DS CHD candidate genes interacted by producing stronger roughened eye phenotypes when co-expressed than when expressed individually or in two copies (e.g., GMR>COL6A1+SH3BGR have highly disorganized eyes with discoloration - Figure 1e). When we considered the aggregate data from over-expressing CHD genes in both the heart and eye, DSCAM and COL6A2 emerged as the most consistently and intensely interacting pair of genes (Figure 1f). We therefore selected this particular combination of genes for further detailed analysis in the hearts of both flies and mice.

In flies, we next examined the basis for the interaction between DSCAM and COL6A2 by taking movies of individual semi-intact fly heart preparations using high speed digital video imaging. We analyzed autonomous heart function using a semi-automated heartbeat analysis that provides several quantitative measures of the dynamic contractile properties of the beating heart [16]. Typically, hearts from young wild-type flies exhibit rhythmic beating patterns with narrow distributions of both diastolic and systolic intervals [13]. We examined dynamic indices of the heart beat in young (one week old) flies expressing both DSCAM and COL6A2 using the heart specific GMH5 driver (GMH5>DSCAM+COL6A2) and found that they exhibited slower and less rhythmic beating than control flies (e.g., non-expressing flies: GMH5/+, DSCAM/+, and COL6A2/+ or flies expressing the transgenes individually (GMH5>DSCAM and GMH5>COL6A2)) (Figure S1a-S1d). In addition, the distribution of the heart period in flies expressing both DSCAM and COL6A2 was substantially broadened (Figure S1b). The increase in heart period in these flies could be attributed to the increase in diastolic interval (Figure S1b-S1d). The alteration of basal heart rate and rhythmicity and disruption of heart function in response to pacing when co-over-expressing DSCAM and COL6A2 indicate that mis-regulation of this particular combination of genes greatly impairs heart function in the fly motivating an analysis of over-expressing these two genes in a mammalian system.

### DSCAM and COL6A2 interact synergistically to cause CHD in mice

**Figure 2:**
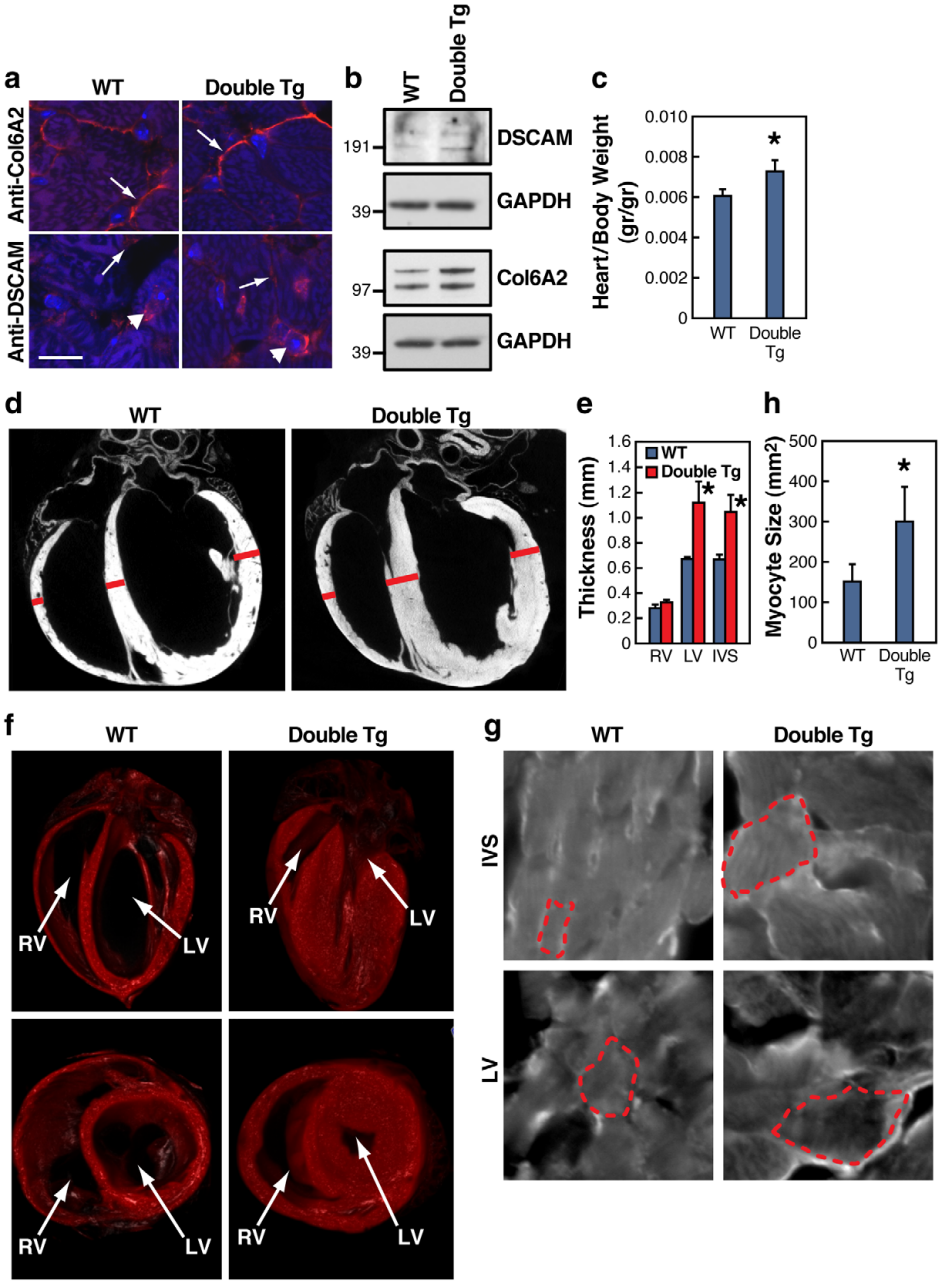
DSCAM and COL6A2 double transgenic mice exhibit cardiac hypertrophy. a) Immunostains of COL6A2 and DSCAM (red) and nuclei (blue) in wild type (WT) and COL6A2 and DSCAM double transgenic mice (Double Tg) in adjacent consecutive sections of 3 month old adult hearts (Bar  =  11 µm). COL6A2 staining is predominantly extracellular (arrows), while DSCAM staining consists of two components; a general plasma membrane surface expression, most notable between adjacent cells (arrows), that would overlap with COL6A2 expression, and intracellular perinuclear staining (arrowhead), which may represent ER-Golgi to plasma membrane transport intermediates of DSCAM. b) Immunoblot analysis of DSCAM and COL6A2 expression in wild-type versus double transgenic mice. GAPDH served as a loading control. c) Increased heart weight in dissected hearts of DSCAM and COL6A2 double transgenic mice (N = 7) compared to their wild-type littermates (N = 5) (± SEM, * t-test (2 tailed, unequal variance) P<0.05). d) Hypertrophy detected by micro-CT in DSCAM and COL6A2 double transgenic hearts. e) Measurements of heart wall thickness (in mm ± SEM) of right ventricle (RV) left Ventricle (LV) and interventricular septum (IVS) derived from Micro-CT virtual sections of double transgenic (N = 8) and wild type (N = 2) hearts (* t-test (2 tailed, unequal variance) P<0.05). Bars in panel d indicate sites of measurement. f) 3-D reconstructions of WT versus double transgenic mouse hearts obtained from micro-CT analysis showing extensive hypertrophy in a frontal section of an adult double transgenic mouse heart. Left ventricle (LV) and right ventricle (RV) are indicated. g) Double transgenic heart myocytes exhibit increased cell size (dotted outlines indicate the borders of individual cells). IVS cardiomyocytes from heart sections stained with fluorescent wheat germ agglutinin. h) Quantification of cell size in IVS from stained heart sections (N = 20, * t-test - 2 tailed, unequal variance, P<0.0001).

Having demonstrated a cooperative interaction between DSCAM and COL6A2 in flies, we next asked whether over-expression of this same pair of genes would similarly result in a synergistic disruption of heart function and/or morphology in mice. We over-expressed each of these genes separately and in combination under the control of the murine heart specific alpha-MHC promoter, which is active in myocardial cells both during early development and in the adult [17]. Single transgenic lines expressing either DSCAM or COL6A2 were fully viable and fertile. Single transgenic lines expressing comparable levels of DSCAM and COL6A2 were chosen and crossed to each other to obtain double transgenic mice co-expressing the two genes in the developing heart. The levels of DSCAM and COL6A2 proteins in transgenic adults were only modestly elevated relative to wild-type (Figure 2a, 2b). In contrast to the full viability of the single transgenic lines (which pertained even to lines expressing considerably higher levels of the transgenes than those used to generate the double transgenic mice), double transgenic adult mice were recovered at only 58% of the expected frequency (Table S3).

Since we targeted expression of the DSCAM and COL6A2 transgenes specifically to the heart, the reduced proportion of viable double transgenic mice suggested that some of these individuals may have succumbed to severe heart defects and that surviving adults might have observable abnormalities in heart morphology or function. Consistent with this possibility, dissected hearts from double transgenic 3-month-old adult mice weighed more than those from wild type controls (Figure 2c). We searched for potential morphological defects using (10 µm) Micro-CT analysis to generate high resolution 3D reconstructions of double transgenic and wild type hearts as well as by inspection of serial heart sections (Figure S2b). The most obvious gross defect we observed in double transgenic hearts was an increased thickness of the left ventricle (LV) wall and interventricular septum (IVS) relative to control wild-type hearts (Figure 2d–2f). In the most extreme double transgenic hearts, digital reconstruction of the heart chambers revealed that the walls of the LV were enlarged to the point of nearly occluding the lumen (Figure 2f). These hearts also had thickened IVS and RV walls. Enlarged left ventricular walls in the hearts of double transgenic mice resemble left ventricular hypertrophic cardiomyopathy that occurs frequently as a result of pressure overload as can result from partial aortic occlusion [18]. Examination of myocyte morphology at the IVS and LV (Figure 2g, 2h) confirmed that the increased thickness of the ventricular walls in the double transgenic animals was a result of increased cell size, as has been observed in various hypertrophic cardiomyopathies [19]. None of these gross morphological defects were observed in wild-type (judged by MicroCT and H&E staining) or single transgenic (by H&E staining) littermates (data not shown).

**Figure 3:**
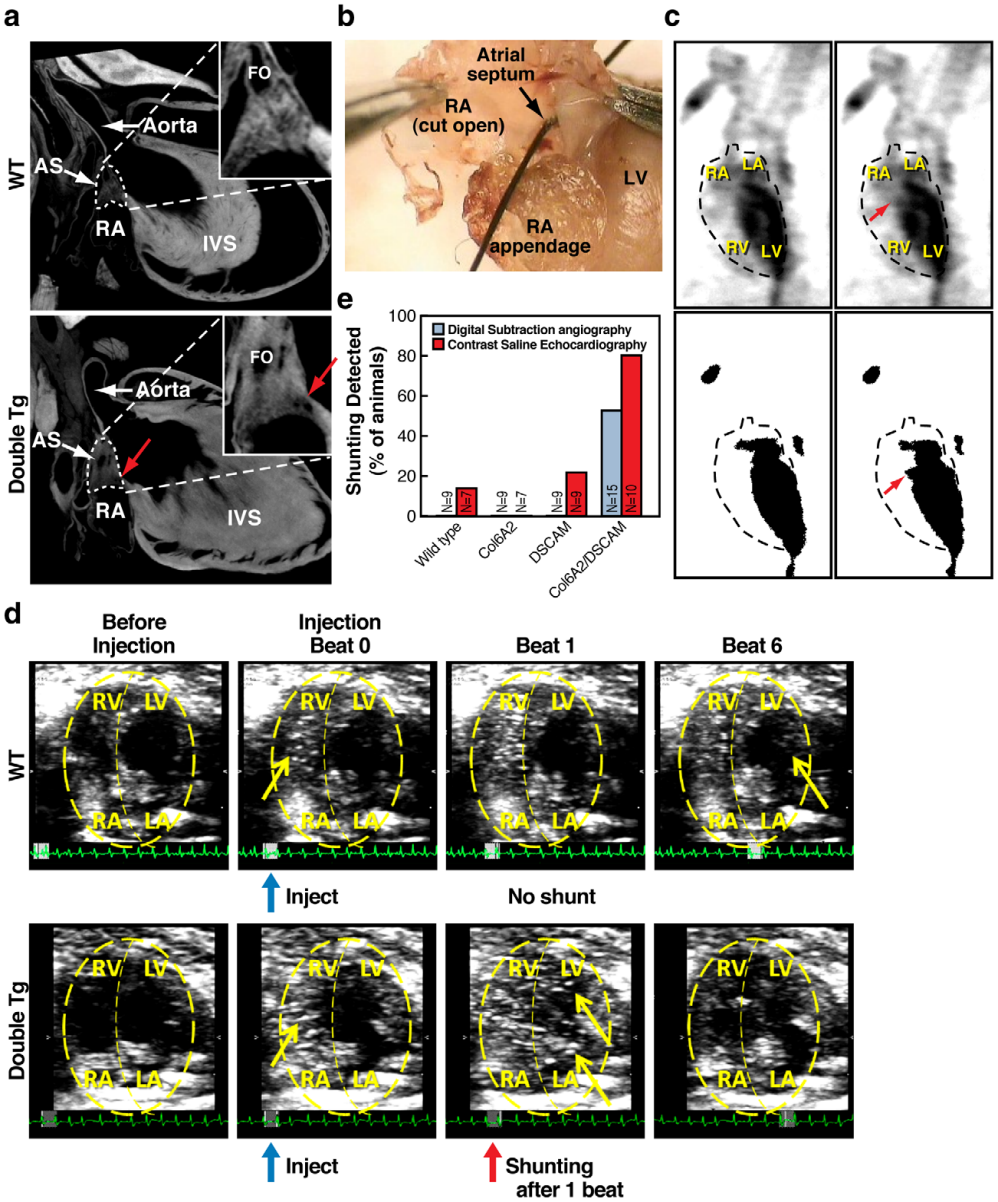
Atrial septal defects in DSCAM and COL6A2 double transgenic mice. a) Atrial Septal Defects (ASD) in a digitally reconstructed double transgenic heart based on Micro-CT imaging. High magnification insets show atrial septal region indicated by dotted lines in the lower magnification full views. Red arrows indicate a hole between left and right atria that also appear on adjacent reconstructed sections indicating that an open passage exists between the two atrial chambers. Atrial septum (AS), right atrium (RA), interventricular septum (IVS), Foramen ovale (FO). b) An ASD in an experimentally dissected double transgenic mouse. A frank hole between the left and right atria allowed the unobstructed passage of a course bristle. c) Digital Subtraction Angiography (DSA) analysis shows shunting of the radio opaque dye from the left atrium to the right atrium. DSA images from double transgenic mouse before (left) and during the detected shunting (right). Top panel-video capture, bottom-binary image after digital subtraction. Red arrows indicate the site of abnormal shunting of the dye from the left atrium into the right atrium. d) Saline contrast echocardiography. Abnormal shunting is detected in double transgenic mice as bubbles in the LV within 1 beat. Panels depict echocardiograms of the cardiac chambers in the axial plane of the heart, both before and after injection of saline. Blue arrows indicate time of injection, red arrows indicate the time bubbles are detected in LV, and yellow arrows indicate bubbles. Each panel consists of 10 frames merged for visualization purposes. e) Percent of wild-type and double transgenic animals exhibiting shunting in digital subtraction angiography and saline contrast echocardiography.

We also examined hearts in greater detail using the full resolution of Micro-CT reconstructive imaging of fixed whole mount hearts as well as standard dissection procedures (Figure 3a, 3b). Both types of fine morphological analysis identified the presence of atrial septal defects (ASDs) in double transgenic mice (N = 7), but not in control wild-type littermates (N = 7). In order to determine whether such frank holes had a physiological consequence and whether such defects were penetrant, we complemented the morphological studies with sensitive physiological measurements monitoring blood flow using two *in vivo* imaging methods. The first method, digital subtraction angiography (DSA), can detect abnormal shunting of blood between the heart chambers by injecting a radio-opaque dye into the right jugular vein and following it through the heart cycle in real-time by radiography [20]. We used DSA analysis to compare the heart function of wild-type, single and double transgenic animals at 3 months of age. We found that 53% of the double transgenic mice tested (N = 15) exhibited abnormal shunting of the dye from the left to the right atrium, indicative of a functional ASD (Figure 3c, 3e). In contrast, none of the wild-type or single transgenic animals displayed any shunting using this assay (Figure 3e, N = 9 for each genotype), demonstrating that this leakage phenotype is fully dependent on both genes being over-expressed.

The second assay we employed, saline contrast echocardiography, provides a yet more sensitive test for shunting in which agitated saline infused with highly reflective micro-bubbles is injected into the right jugular vein and then followed by 2-dimensional echocardiography. During the period when the pressure in the right atrium exceeds that in the left atrium, even minute amounts of right-to-left blood flow shunts can be detected [21], [22]. In this case, we observed a yet higher frequency of shunting from the right to left atrium in double transgenic mice (80%, N = 10) (Figure 3d, 3e, and Videos S1 and S2). We also observed shunting in one wild-type mouse (14%, N = 8) and in two single DSCAM transgenic mice, (22%, N = 9), but not any of the single COL6A2 transgenic mice (N = 7). For several double transgenic mice that displayed atrial shunting by echocardiography (N = 6), we performed additional morphological analysis by serial sectioning of the hearts. These individuals all displayed multiple defects in the atrial septum as well as septal dysmorphologies (e.g., Figure S2). We conclude both by functional and morphological criteria that double, but not single, transgenic mice have a high frequency of functional atrial septal defects.

### DSCAM and COL6A2 cooperatively promote substrate adhesion

**Figure 4:**
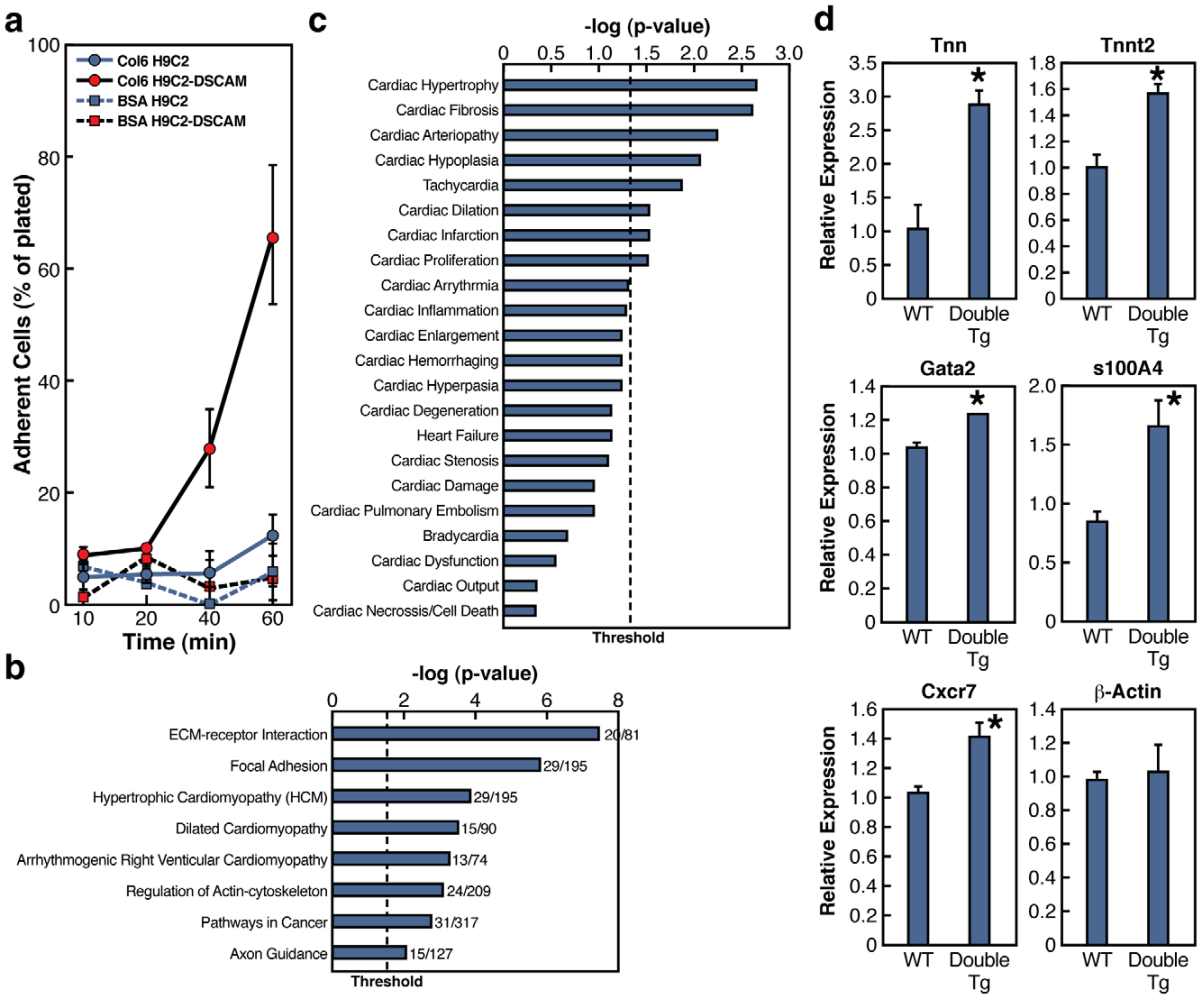
DSCAM and COL6A2 enhance cell adhesion and induce transcriptional changes. a) Cell adhesion assay in a cardiomyocyte cell line. A stable line of H9C2 cardiomyocyte cells expressing DSCAM (H9C2-DSCAM) or parental H9C2 cells were grown on BSA or COL6 coated plates. At the indicated time points, plates were gently washed and the number of cells retained on the plates was determined shown as percent of cells adhering to plates. b) The top gene ontology KEGG pathway terms of differentially expressed genes identified by RNA-seq of H9C2 and H9C2-DSCAM cell lines grown on COL6 coated plates. Threshold shown p<0.05. c) Enriched heart related gene ontology terms in H9C2 vs. H9C2-DSCAM differentially expressed mRNAs using Ingenuity knowledge pathway database. Threshold shown p<0.05. d) qRT-PCR of select genes in wild type and double transgenic e14.5 hearts. Results shown as average +/− SEM (N = 3). Unpaired two-tailed t-test analysis p<0.05 (*).

DSCAM is a cell adhesion molecule and its genetic interaction with COL6A2, an extracellular matrix component, suggested a possible direct or indirect interaction involving cell-substrate adhesion. We investigated this possibility by transfecting the rat cardiac myoblast cell line H9C2 with DSCAM to generate a stable cell line constitutively expressing DSCAM (H9C2-DSCAM). These cells moderately over-express DSCAM and exhibit a subcellular distribution that is similar to the endogenous protein (Figure S3). We plated these DSCAM-expressing cells or non-transfected H9C2 control cells on microtiter wells coated with COL6 or BSA to assay substrate adhesion. We observed that H9C2-DSCAM cells adhered more firmly to the COL6 substrate than non-transfected cells in a time dependent fashion (Figure 4a).

Since H9C2-DSCAM cells exhibited elevated adhesion in a COL6-dependent fashion and because adhesive interactions are known to induce transcriptional responses, we asked whether expression of DSCAM in H9C2 cells might alter the transcriptional profile of these cells. We purified RNA from H9C2-DSCAM and control H9C2 cells plated on COL6 and performed RNA deep sequencing (RNA-Seq) and compared the transcriptomes of the DSCAM-expressing and parental cell lines. Our analysis identified 1251 genes that were differentially expressed in the two cell lines (Dataset S1). Gene ontology (GO) analysis of genes that were changed more than 2-fold in DSCAM-expressing versus parental cells (p<0.0005) identified genes involved in cellular adhesion (GO terms: ECM-Receptor interaction and focal adhesion - Figure 4b) and cardiomyopathies (GO: hypertrophic cardiomyopathy, cardiac hypertrophy, fibrosis - Figure 4c and Table S5), which is consistent with the prominent cardiac hypertrophy we observed in double transgenic animals expressing DSCAM and COL6A2 (Figure 4b and Table S4). In line with the bulk analysis of GO terms, we found that a number of genes that promote adhesion were up-regulated including collagens, cadherins and integrins (e.g. COL6A1, COL4A1, COL18A1, CDH3), whereas genes associated with migration or metastatic tumor invasion were down-regulated (e.g. TWIST1, TIAM1, SLIT3, STAT1/STAT5A, BMP2). In summary, the whole genome transcriptional analysis supports the notion that co-expression of the DSCAM and COL6A2 genes results in a transcriptional misregulation of genes involved in cell-cell adhesion and ECM-cell interaction, which may contribute to the observed increased adhesion of H9C2-DSCAM cells to collagen substrate, as well as genes that respond transcriptionally in patients with cardiomyopathies.

The altered expression levels of genes mediating cell-substrate interactions or genes misregulated in cardiac myopathy and hypertrophy in H9C2-DSCAM/COL6 cardiomyocyte cells suggested that these genes might also be misregulated *in vivo* in double transgenic DSCAM+COL6A2 mice. We therefore examined the relative expression levels of a select set of genes in hearts dissected from wild-type and double transgenic embryos. mRNA was isolated from individual hearts and tested for quantitative changes in gene expression by qRT-PCR. This analysis confirmed that several gene transcripts altered in the cellular cardiomyocyte model were also affected in the hearts of the double transgenic mice by qRT-PCR (Figure 4d), including those encoding focal adhesion protein tenascin N (*Tnn*), hypertrophy associated cardiac troponin gene (*Tnnt2*), calcium binding protein *S100A4*, which is involved in fibrosis and tissue remodeling in several diseases [23], *Cxcr7*, which is associated with various cardiac defects including septal defects [24], [25], and the transcription factor *Gata2*, which is associated with familial early-onset coronary artery disease [26]. Taken together, the expression results from the cardiomyocyte and transgenic mice hearts suggest that increased DSCAM and COL6A2 expression induce a transcriptional response that could amplify excessive adhesion and contribute to heart malfunction.

## Discussion

### DSCAM and COL6A2 cooperatively disrupt heart function in flies and mice

In this study, we began with information provided by decades of mapping in human DS patients that delimited a small region of chromosome 21 responsible for causing CHD [6]-[10]. Expression data indicating which genes in this interval were expressed in the heart further restricted the set of potential candidate genes that might contribute to CHD. A contributory role of DSCAM has also been proposed [9], but given the large numbers of subjects needed for fine genetic mapping it would have been very difficult to go much beyond this level of analysis using human genetic data alone. We therefore turned to the fly as a model system with a beating heart tube that shares many basic cell biological features of the mammalian heart to identify stringent forms of genetic interaction associated with over-expression of CHD candidate genes in all possible pairwise combinations. This comprehensive first-order analysis of basic heart function indices pointed to two genes, DSCAM and COL6A2, as causing the most severe synergistic disruption of heart function in flies. Further in depth analysis using quantitative real-time imaging of fly hearts over-expressing DSCAM and/or COL6A2 revealed additional cooperative defects such as arrhythmicity. While the interaction between these two genes was the strongest, we note that there were also fairly strong interactions between DSCAM and two other genes, COL6A1 and SH3BGR. The interaction of DSCAM with COL6A1 is not too surprising given that its gene product and that of COL6A2 are subunits of a common tripartite helical collagen fiber. The interaction with SH3BGR warrants further scrutiny in future studies, however, as it may provide a link between the extracellular compartment and signal transmission into cardiac cells.

Because DSCAM and COL6A2 cooperatively altered heart function in flies when expressed in myocardial cells, we selected this combination of genes to over-express at modest levels in the murine heart. We chose the myosin-alpha chain gene promoter to drive over-expression of these genes in the myocardium since it is heart specific and drives modest levels of expression both during heart development and in adult hearts. While this mode of expression obviously does not precisely recapitulate the endogenous pattern of DSCAM and COL6A2 expression, it does restrict expression of these genes to the heart throughout a protracted period. Since we were using this driver to express molecules targeted to the extracellular space, we reasoned that these proteins might similarly accumulate extracellularly whether they were expressed by cardiomyocytes or other cardiac cells such as fibroblasts that are known to produce these proteins as well (note that since both DSCAM and COL6A2 are expressed in developing fetal myocytes, we recapitulated at least a subset of the endogenous expression pattern). Additional studies with endogenous promoters or promoters driving expression of these genes in other cell populations in the heart (fibroblasts or neural crest derivatives) are clearly warranted. The choice of gene expression vehicle notwithstanding, we observed a very strong synergistic interaction between DSCAM and COL6A2 when over-expressed in the mouse heart including, highly penetrant and prominent cardiac hypertrophy, atrial septal defects associated with physiological shunting, and a mortality rate of 50%. Most importantly, these dramatic phenotypes were not observed in transgenic mice expressing either transgene alone, but only in mice co-expressing both transgenes.

### DSCAM and COL6A2 cooperatively promote cell-substrate adhesion

An obvious possible mechanism by which DSCAM and COL6A2 might disrupt heart development and/or function is by altering cell-substrate adhesion given that DSCAM is a transmembrane adhesion molecule and COL6A2 is constituent of the ECM. In line with this possibility, a rat cardiomyocyte cell line expressing DSCAM exhibits a time-dependent increased adhesion to COL6 coated plates. Although it is known that adhesion of cells has transcriptional effects, we were surprised that much of this transcriptional response seems to be focused on regulation of genes involved in the adhesion network, suggesting that positive feedback mechanisms further stabilizing adhesive interactions may be a prominent element of this interaction. These pervasive changes in gene expression could be mediated either by DSCAM itself, which is known to transduce a variety of signaling events during axonal pathfinding and axon branching including self-adhesive signaling and a response to Netrins, or by other adhesion dependent effectors such as the focal adhesion pathway components of which are regulated in the transcriptional response of H9C2-DSCAM grown on COL6. The reciprocal regulation of several components promoting adhesion versus cell migration may also contribute to the CHD phenotypes that are observed in DSCAM+COL6A2 double transgenic mice, particularly since a number of the responsive genes identified in the cell culture experiments were also misregulated in hearts of double transgenic mice. While further studies will be needed to assess the importance of altered cell-substrate adhesion and cell migratory processes in mediating the morphological and physiological effects of DSCAM+COL6A2 over-expression, we speculate that such defects could lead to a developmental delay in closing the atrial septum and may also contribute, either developmentally or as part of a physiological feedback loop, to hypertrophic phenotypes that we observed in affected mice.

### Potential relevance of CHD in double transgenic mice to human cardiac disease

Since we set out to identify genes contributing to DS CHD, a natural question is whether the phenotypes we observe recapitulate those associated with DS in humans. It is certainly noteworthy that we observed ASDs with high penetrance in double transgenic mice, since this is one of several salient features of DS CHD. However, other typical DS CHD phenotypes were not observed such as atrial ventricular septal defects, perimembranous and muscular ventricular septal defects, Tetralogy of Fallot, or persistent ductus arteriosus. Also, since atrial septal defects can have many etiologies, in order to determine whether this phenotype is similar to that in human DS patients, it will be important to examine in greater detail the origin of these defects in mice (e.g., premium or secundum ASD) and compare them to the occurrence of these defects in DS patients [27].

There are several possible reasons for DSCAM+COL6A2 double transgenic mice exhibit only a subset of DS CHD phenotypes. Perhaps most obviously, as noted above, endogenous promoters may drive expression of these genes at different levels or in distinct spatial and temporal patterns than we achieved using a heterologous promoter. With regard to expression level, however, we note that DSCAM+COL6A2 double transgenic mice express only modestly elevated levels of the transgenes. Whether these match precisely with the altered dose in DS patients is unknown. It is noteworthy in this context that the level of altered gene expression in DS patients is not always elevated by precisely 50%, and in some cases can be as much as 2-3 times the normal level [28], [29], as is the case for COL6A2 in human DS brains [29]. Another factor to consider is that in our studies we focused on phenotypes caused by over-expressing DSCAM and COL6A2 in myocardial cells. However, some of the AV septal complex formation relies on endocardium and the endocardial derived mesenchyme, which undergoes complex remodeling processes in the AV cushion region [30], and thus would not be targeted by the transgenic promoter we utilized. Moreover, migratory neural crest derivatives of the heart as well as cardiac fibroblasts, which have been implicated in cardiac hypertrophy [31], may also play important roles in DS CHD. In addition, there may be species specific differences in response to altered gene expression levels. For example, mice carrying a complete copy of human chromosome 21 (Tc1 mice) do not reproduce the full spectrum of DS CHD defects observed in humans [32], [33]. Similarly, trisomy of mouse chromosome 16, which includes most but not all murine orthologues of genes carried on human chromosome 21, results in abnormal atrioventricular junction defects that are not present in human DS CHD [34]. Finally, DS CHD may involve the over-expression of other genes in addition to DSCAM and COL6A2. Since DSCAM also interacted strongly with SH3BGR in flies, it would be of particular interest to examine the consequence of over-expressing this pair of genes in mice.

The most prominent phenotype we observed in DSCAM+COL6A2 double transgenic mice was pronounced left ventricular hypertrophic cardiomyopathy, which is not typical of DS CHD [35], [36]. The basis for this non-DS phenotype may be the same as those responsible for only a partial recapitulation of the DS phenotype such as level, timing, or pattern of transgene expression. The exact basis for this hypertrophic phenotype notwithstanding, it is highly penetrant in double transgenic mice. Since this phenotype, like ASD, is only observed in double transgenic mice and not in the single transgenic strains, this genetic interaction may be highly relevant to the etiology of various forms of cardiac hypertrophy such as those resulting from increased load [18], [19], since coordinate up-regulation of these genes that may occur spontaneously as a result of somatic mutation or epigenetic responses, may generate similar phenotypes in humans. Further analysis will be required to determine whether DSCAM and COL6A2 contribute to ASD typical of DS and whether levels of these two genes are jointly increased in patients with inherited or spontaneous forms of cardiac hypertrophy.

### The strength of a multi-tiered genetic analysis

Success in identifying DSCAM and COL6A2 as mediators of CHD phenotypes in these studies resulted from the combined use of three systems: comprehensive candidate testing in flies, validation of synergistic genetics interactions in mice and cell culture, and high resolution genetic mapping in humans. In addition to these genetic studies, we then employed cell based assays to investigate the underlying molecular mechanisms regulated by these gene products. For this initial study, we restricted our screen to genes that we suspected had a high chance of causing heart defects based on *a priori* assumptions including genetic analysis in human DS patients, expression in the developing heart, and the potential to physically interact in the extracellular environment. While these criteria naturally limit our analysis to a subset of possible contributing genes, they nonetheless lay the groundwork for future studies to analyze contributions of additional genes from the CHD candidate region.

We suggest that application of this multi-tiered genetic strategy should be broadly applicable to other multigenic genetic diseases in which sorting though many genetic combinations is necessary to identify promising candidate loci underlying disease phenotypes. Examples of such multigenic disorders include the identification of interacting genes within loci defined by the HapMap initiative [5], contiguous gene disorders associated with macroscopic duplication or deletion syndromes such as those underlying autism [37], and potentially a large number of spontaneous as well as heritable conditions resulting from alterations in gene dose due to CNVs, which have been identified throughout the human genome [4]. These data also imply that extracellular proteins can exert potent effects on gene transcription programs as a component of their phenotypic effects, raising intriguing mechanistic questions for future investigation.

## Methods

### Fly stocks and DNA clones

All fly stocks were raised at 25 °C. Full-length cDNAs were mis expressed in the heart or developing eye using the GAL4-UAS transactivation system [14]. Full-length mammalian cDNAs of SH3BGR (NM_007341), DSCAM (NM_031174), COL6A1 (NM_001848) and COL6A2 (NM_001849) have been described previously [10]. *Drosophila* full-length cDNA of SH3β (CG8582) and COL18A1 (CG33171) were obtained from Open Biosystems. The mammalian and *Drosophila* cDNAs were cloned into pUAS vector and injected to w^1118^ embryos. Several independent transgenic lines were generated by *P*-element-mediated germ line transformation technique for each *pUAS* construct. GMR-GAL4 Stock was obtained from Bloomington, Indiana. The GMH5-GAL4 line [15] was used to drive the expression of DS CHD candidate genes specifically to the myocardial cells in the heart.

### Fly heart physiology

Heart rate, electrical pacing, semi intact heart preparation and movie analysis were conducted as described previously [15], [16].

### Digital subtraction angiography

250 µL of nonionic contrast was injected into the jugular vein over a period of 1 to 2 seconds and video images were acquired on half-inch super-VHS videotape at 30 frames per second under constant fluoroscopy with the XiScan 1000 C-arm x-ray system (XiTec, Inc; 3-inch field of view, anterior-posterior, lateral and left anterior oblique projections). Later, the interlaced video images were edited and digitally processed off-line (Silicon Graphics R10000 system, Motif 6.5 operating system) with a resolution matrix of 512x512 pixels, 256 shades of gray, 60 fields per second [20].

### Saline contrast echocardiograms

Mice were sedated with isoflurane. The jugular vein was dissected and cannulated for the intravenous saline administration. Imaging of the heart was performed using an Echo ultrasound system. Image acquisition in the axial four-chamber views was begun just before injection of contrast and continued until contrast effect in the myocardium had dissipated [21], [22]. Detection of contrast in the LA and LV within 1-2 heart beats following injection of contrast saline was an indication of an abnormal shunting. Occasionally contrast was detected in the LV after 6 or more heart beats due to residual contrast recirculation through the lungs, and was not considered an indication of shunting.

### MicroCT analysis

High-resolution volumetric Computed Tomography (CT) of hearts was performed by Numira Biosciences (Irvine, CA) at 10-µm^3^ isometric voxel resolution using an eXplore Locus SP MicroCT specimen scanner (GE Healthcare, London, Ontario, Canada). Visualization of sections was performed with MicroView Software (GE healthcare) and volume rendering was performed with OsiriX Medical Image software.

### Immunohistochemistry

Hearts were cut at the horizontal short-axis plane, fixed in 4% paraformaldehyde, embedded in OCT and sectioned. Frozen cryosections wild-type and double transgenic mice were stained with anti DSCAM (N-16) (Santa Cruz biotechnology) or anti COL6A2 (D20) (Santa Cruz biotechnology), and anti Rabbit Alexa594 secondary (Jackson immunochemicals), counter stained with Hoechst 33342 (Invitrogen) imaged with a Perkin Elmer UltraView Vox spinning disk confocal microscope. For myocytes size determination, 14 micron frozen cryosections were stained with Alexa 594 –conjugated wheat germ agglutinin (Invitrogen), and the myocyte cross-sectional area was measured for assessment of cell size using NIH image J software. For staining of H9C2 and H9C2-DSCAM cells, cells were grown on 35 mm plates, fixed in 4% paraformaldehyde, permeabilized in PBS/Triton X100 0.1%, and stained with rabbit anti DSCAM (a kind gift from Dr. Elke Stein, Yale University, CT), or mouse anti Myc (9E10, Santa Cruz biotechnology) primary antibodies, Alexa594 secondary antibodies (Jackson immunochemicals), counter stained with Hoechst 33342 (Invitrogen) and imaged with a Zeiss Axioplan 2 fluorescence microscope.

### Generation of the H9C2-DSCAM cell line

DSCAM was cloned in to an expression vector expressing a C-terminal myc tag and a puromycin resistance gene. The promoter used was a modified chicken beta actin promoter which can be expressed in a wide variety of tissues and cell types (pCAGGS promoter). H9C2 cells were transfected and selection resistant clones were isolated. We chose a cell line that over-expressed DSCAM at the lowest level that we could still detect tagged DSCAM by western blotting.

### Cell adhesion assay

Cell adhesion was performed as described [38]. Briefly, 96 well plates were coated with 10 µg/ml COL6 (Meridian life sciences, ME) in PBS (-Ca^++^, -Mg^++^) overnight, washed twice with PBS (-Ca^++^, -Mg^++^), and then incubated for 2 hours with 1% BSA/PBS (-Ca^++^, -Mg^++^) to block non-specific binding. In control (BSA) plates addition of collagen was omitted and plates were processed in parallel. 2.5×10^5^ cells/ml H9C2 or H9C2-DSCAM cells were plated in serum free media on blocked 96 well plates in triplicate. At the indicated time points, wells were washed twice gently in serum free media, and attached cells were fixed in 10% neutral buffered formalin for 5 minutes. Cell number was determined by crystal violet staining, read at OD^540^. The percent of cells bound was calculated relative to cells plated in non-blocked wells which displayed 100% adherence at the end point of the experiment.

### mRNA sequencing and analysis

#### Library preparation for Illumina sequencing

Poly-T capture beads were used to isolate mRNA from 5 µg of total RNA from either H9C2 or H9C2-DSCAM cells grown on COL6 coated plates. First-strand cDNA was generated using random hexamer-primed reverse transcription, and subsequently used to generate second-strand cDNA using RNase H and DNA polymerase. Sequencing adaptors were ligated using the Illumina Genomic DNA sample prep kit. Fragments ∼200 bp long were isolated by gel electrophoresis, amplified by 16 cycles of PCR, and sequenced on the Illumina Genome Analyzer II.

#### Computational analyses of mRNA-Seq read data

Sequence reads were filtered for quality, failing tags with more than 8Ns or 8 bases with quality values lower than 3 standard deviations from the mean. Tags were then aligned using the top hat splice read mapper [39]. Cufflinks [40] was then used to calculate the “FPKM” transcript expression levels using the refSeq annotations from the rn4 freeze available through the UCSD genome browser. Homologous mouse mm8 genes were taken from the rn4 xenoRefGene table to fill in areas not covered by the rn4 refSeq track. The resulting transcripts were filtered under the criteria: p-value<0.00005, FPKM.min≥2, fold change ≥2. The list of significantly changed genes was then analyzed for Gene Ontology term enrichment by implementing the Database for Annotation, Visualization, and Integrated Discovery (DAVID) Gene Ontology (GO) search engine (http://david.abcc.ncifcrf.gov/) [41], [42]. Functional Analysis of RNA-seq data set using Ingenuity Pathways Knowledgebase: The Functional Analysis identified the biological functions and/or diseases that were most significant to the data set. Molecules from the dataset that were associated with biological functions and/or diseases in Ingenuity's Knowledge Base were considered for the analysis. Right-tailed Fisher's exact test was used to calculate a p-value determining the probability that each biological function and/or disease assigned to that data set is due to chance alone.

## Supporting Information

Dataset S1 H9C2-DSCAM mRNA-seq data.
(XLS)

Figure S1 DSCAM and COL6A2 over-expression cause contractility defects in the fly heart. a) Representative 10 second M mode traces extracted from high speed movies of semi intact 1 week old flies. The genotypes tested were the heart specific GAL4 driver GMH5 alone (GMH5/+), flies carrying one or both the DSCAM and COL6A2 transgenes without GAL4 driver (DSCAM/+, COL6A2/+ and both), or flies carrying both the GMH5-GAL4 driver and one or both transgenes (GMH5>DSCAM, GMH5>COL6A2 and GMH5>DSCAM+COL6A2). b-d) Histograms of Heart beat parameters distribution at 1 week old flies (N = 20), of heart period (b), diastolic interval (c) and systolic interval (d).
(TIF)

Figure S2 Documenting atrial shunting and atrial septal defects in the same heart of a DSCAM/COL6A2 double transgenic mouse. a) Saline contrast echocardiography showing abnormal shunting detected as bubbles that appear within 1 beat in the LV. Panels depict echocardiograms of the cardiac chambers in the axial plane of the heart, both before and after injection of saline. Blue arrows indicate time of injection, red arrows indicate the time bubbles are detected in LV, and yellow arrows indicate bubbles. Each panel consists of 10 frames merged for visualization purposes. b) Unstained serial cryosections of the same DSCAM/COL6A2 double transgenic heart that exhibited abnormal shunting as shown in (a) sectioned at 14 µm. The sections reveal frank holes in the atrial septum within the foramen ovale (red arrows) as well as dysmorphology of the atrial septum (green arrows), which may contribute to a fenestrated septum. Consecutive sections are shown at the atrial septum level, from the apex towards the base of the heart (panels- left to right, top to bottom). Atrial septum (AS), right atrium (RA), left atrium (LA) right ventricle (RV), left ventricle (LV), tricuspid valve (TV), mitral valve (MV).
(TIF)

Figure S3 Expression of DSCAM in H9C2-DSCAM cells. a) Western blot of H9C2 and H9C2-DSCAM cells showing the relative expression level of DSCAM using a rabbit anti-DSCAM antibody, showing moderate increased level of expression in the H9C2-DSCAM cells relative to control H9C2 cells. b) Immunostaining using rabbit anti-DSCAM antibody showing increased expression in H9C2-DSCAM cells, mainly in perinuclear (Golgi/ER) (arrow) and membrane regions (arrowhead) (DSCAM – red, nuclei – blue). c) Immunostaining against the Myc tagged DSCAM identifies both perinuclear (arrow) as well as membrane staining, especially in areas between adjacent cells (arrowhead) (Myc – Green, nuclei – blue).
(TIF)

Table S1 Human DS CHD candidate genes and their *Drosophila* homologs. Asterisk (*) indicates DS CHD fly and mammalian genes used to generate transgenic lines).
(DOC)

Table S2 Summary of heart performance parameter changes in flies expressing all candidate CHD genes in single and pairwise combinations. The three asterisks (***) indicate statistically significant differences from the corresponding control (Chi square, P<0.05) and a minus (-) indicates no significant difference from the control.
(DOC)

Table S3 Transmission frequencies of DSCAM and COL6A2 transgenes in mice. For double transgenic mice, the frequency of transmission is reduced to 58% of the expected rate (Chi square, P = 1.0904E-07).
(DOC)

Table S4 List of Gene Ontology KEGG pathway genes.
(DOC)

Table S5 Genes associated with cardiomyopathy.
(DOC)

Video S1 Saline contrast echocardiography movie of 3 month old wild-type mouse.
(AVI)

Video S2 Saline contrast echocardiography movie of 3 month old DSCAM and COL6A2 double transgenic mouse.
(AVI)
