# Supplementary figures and images for: Over-Expression of DSCAM and COL6A2 Cooperatively Generates Congenital Heart Defects
Source: PLoS Genet. 2011 Nov 3;7(11):e1002344. doi: 10.1371/journal.pgen.1002344 (PMC3207880; doi:10.1371/journal.pgen.1002344)

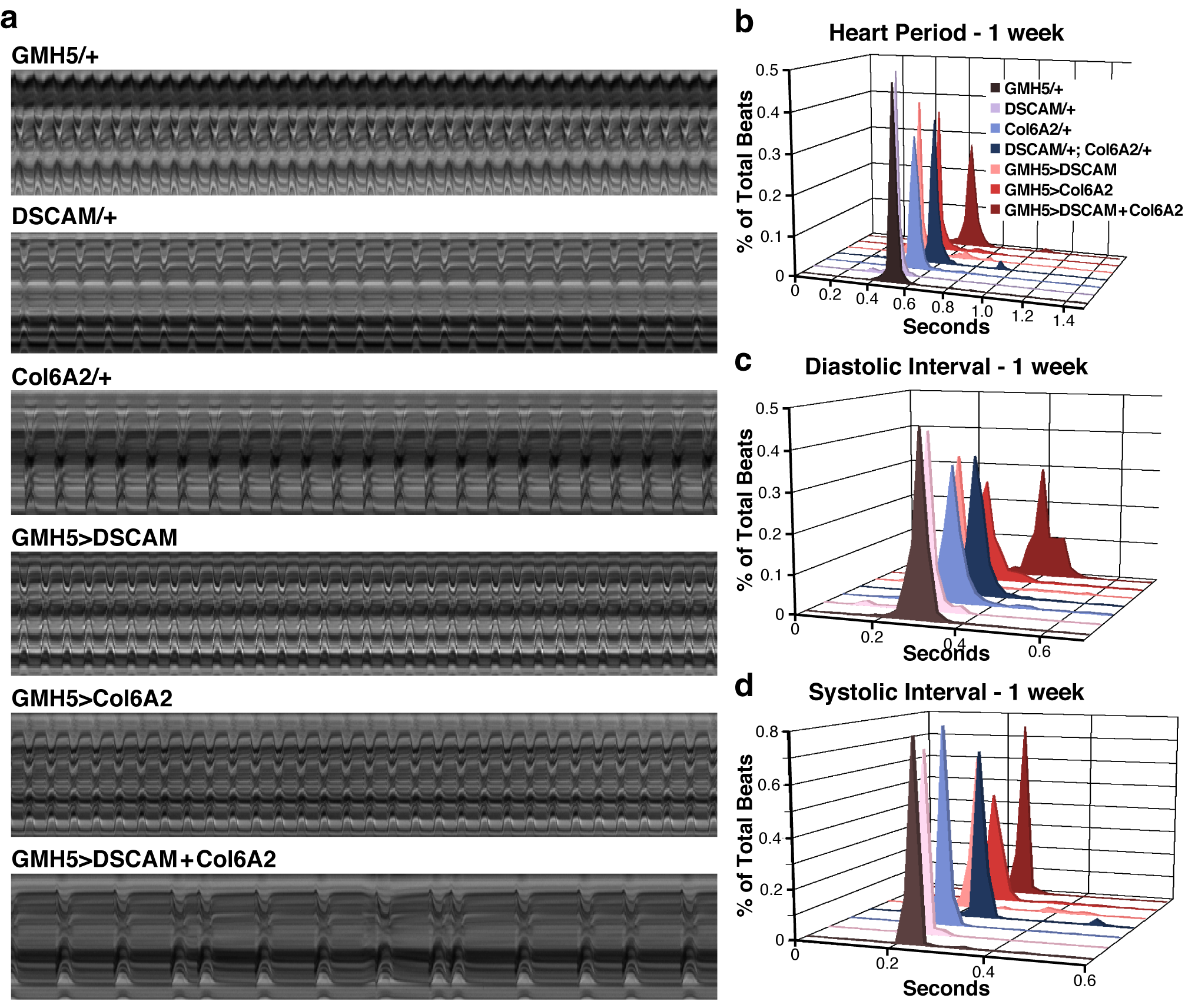

Supplement: Figure S1 — DSCAM and COL6A2 over-expression cause contractility defects in the fly heart. a) Representative 10 second M mode traces extracted from high speed movies of semi intact 1 week old flies. The genotypes tested were the heart specific GAL4 driver GMH5 alone (GMH5/+), flies carrying one or both the DSCAM and COL6A2 transgenes without GAL4 driver (DSCAM/+, COL6A2/+ and both), or flies carrying both the GMH5-GAL4 driver and one or both transgenes (GMH5>DSCAM, GMH5>COL6A2 and GMH5>DSCAM+COL6A2). b-d) Histograms of Heart beat parameters distribution at 1 week old flies (N = 20), of heart period (b), diastolic interval (c) and systolic interval (d). (TIF) [file pgen.1002344.s002.tif]

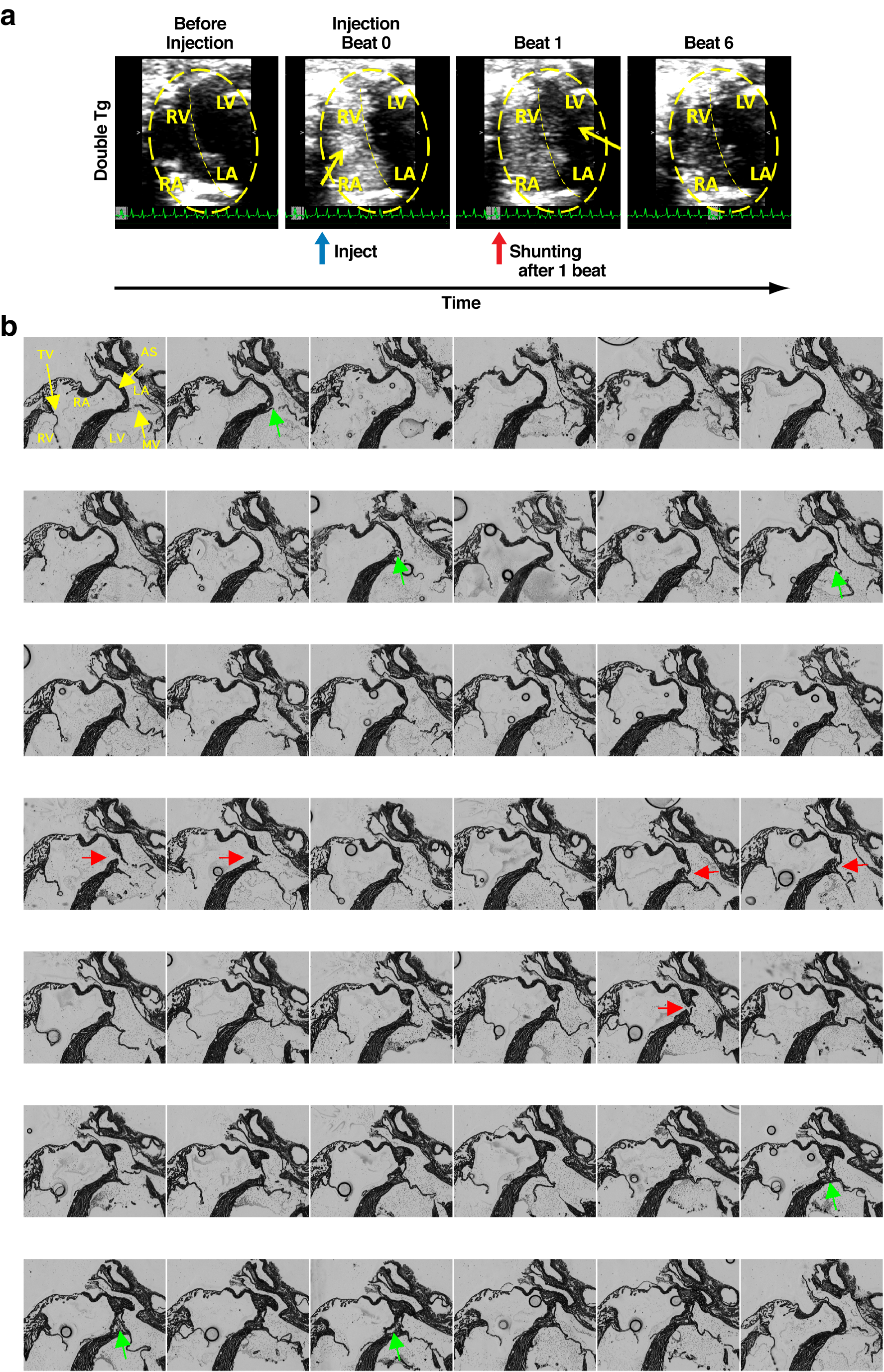

Supplement: Figure S2 — Documenting atrial shunting and atrial septal defects in the same heart of a DSCAM/COL6A2 double transgenic mouse. a) Saline contrast echocardiography showing abnormal shunting detected as bubbles that appear within 1 beat in the LV. Panels depict echocardiograms of the cardiac chambers in the axial plane of the heart, both before and after injection of saline. Blue arrows indicate time of injection, red arrows indicate the time bubbles are detected in LV, and yellow arrows indicate bubbles. Each panel consists of 10 frames merged for visualization purposes. b) Unstained serial cryosections of the same DSCAM/COL6A2 double transgenic heart that exhibited abnormal shunting as shown in (a) sectioned at 14 µm. The sections reveal frank holes in the atrial septum within the foramen ovale (red arrows) as well as dysmorphology of the atrial septum (green arrows), which may contribute to a fenestrated septum. Consecutive sections are shown at the atrial septum level, from the apex towards the base of the heart (panels- left to right, top to bottom). Atrial septum (AS), right atrium (RA), left atrium (LA) right ventricle (RV), left ventricle (LV), tricuspid valve (TV), mitral valve (MV). (TIF) [file pgen.1002344.s003.tif]

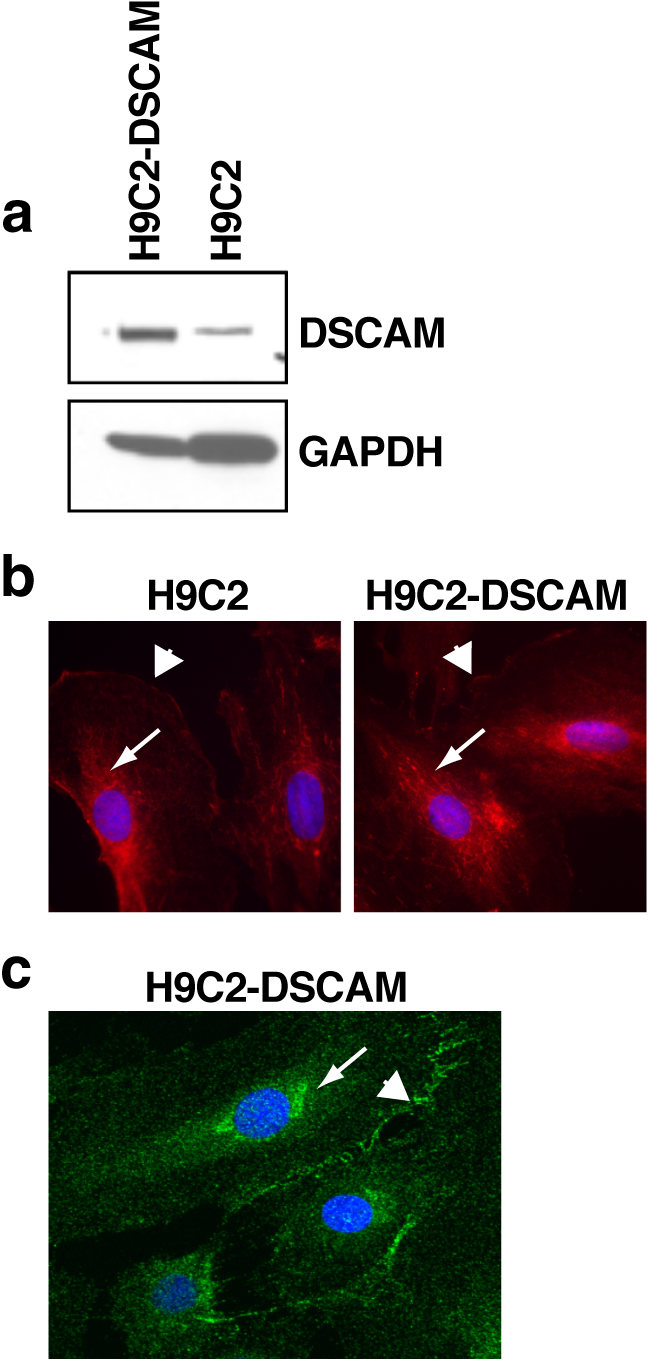

Supplement: Figure S3 — Expression of DSCAM in H9C2-DSCAM cells. a) Western blot of H9C2 and H9C2-DSCAM cells showing the relative expression level of DSCAM using a rabbit anti-DSCAM antibody, showing moderate increased level of expression in the H9C2-DSCAM cells relative to control H9C2 cells. b) Immunostaining using rabbit anti-DSCAM antibody showing increased expression in H9C2-DSCAM cells, mainly in perinuclear (Golgi/ER) (arrow) and membrane regions (arrowhead) (DSCAM – red, nuclei – blue). c) Immunostaining against the Myc tagged DSCAM identifies both perinuclear (arrow) as well as membrane staining, especially in areas between adjacent cells (arrowhead) (Myc – Green, nuclei – blue). (TIF) [file pgen.1002344.s004.tif]
