# Supplementary material for: Over-Expression of DSCAM and COL6A2 Cooperatively Generates Congenital Heart Defects
Source: PLoS Genet. 2011 Nov 3;7(11):e1002344. doi: 10.1371/journal.pgen.1002344 (PMC3207880; doi:10.1371/journal.pgen.1002344)
Supplement: Table S1 — Human DS CHD candidate genes and their Drosophila homologs. Asterisk (*) indicates DS CHD fly and mammalian genes used to generate transgenic lines). (DOC) [file pgen.1002344.s005.doc]

**Supporting Table S1:**

| **Human Gene** | **Disease** | **Fly Gene** | **Function** | **Expression in  fly embryo** |
| --- | --- | --- | --- | --- |
| **SH3BGR*** | Down Syndrome | dSH3β CG8582* | Adaptor protein | Ubiquitous |
| **DSCAM*** | Down Syndrome | Dscam  CG17800 | Axon guidance receptor | Nervous system, Heart |
| **COL6A1*** | Down Syndrome,  Bethlem Myopathy,  Ulrich Congenital Muscular Dystrophy | Dcg1 CG4145  Vkg CG16858 | Collagen type VI α1 subunit | Ubiquitous |
| **COL6A2*** | Down Syndrome,  Bethlem Myopathy,  Ulrich Congenital Muscular Dystrophy | Dcg1 CG4145  Vkg CG16858 | Collagen type VI α2 subunit | Ubiquitous |
| **COL18A1** | Down Syndrome,  Knobloch Syndrome | dCOL18A1  CG33171* | Collagen type XVIII α1 subunit | Heart specific |
