# Supplementary material for: Over-Expression of DSCAM and COL6A2 Cooperatively Generates Congenital Heart Defects
Source: PLoS Genet. 2011 Nov 3;7(11):e1002344. doi: 10.1371/journal.pgen.1002344 (PMC3207880; doi:10.1371/journal.pgen.1002344)
Supplement: Table S2 — Summary of heart performance parameter changes in flies expressing all candidate CHD genes in single and pairwise combinations. The three asterisks (***) indicate statistically significant differences from the corresponding control (Chi square, P<0.05) and a minus (-) indicates no significant difference from the control. (DOC) [file pgen.1002344.s006.doc]

**Supporting Table S2:**

Single transgenic

|  | **Heart rate** | **Failure rate** | **Recovery rate** |
| --- | --- | --- | --- |
| **GMH5 > DSCAM** | ******* | ******* | **-** |
| **GMH5 > DSCAM (x2)** | ******* | ******* | **-** |
| **GMH5 > Col6A2 (x2)** | **-** | ******* | ******* |
| **GMH5 > dSH3b (x2)** | ******* | **-** | ******* |
| **GMH5 > Col6A2** | **-** | ******* | ******* |
| **GMH5 > Col6A1** | **-** | ******* | **-** |
| **GMH5 > Col6A1 (x2)** | **-** | ******* | **-** |
| **GMH5 > SH3BGR** | **-** | ******* | **-** |
| **GMH5 > SH3BGR (x2)** | **-** | ******* | **-** |
| **GMH5 > dSH3b** | **-** | **-** | **-** |
| **GMH5 > Col18A1** | **-** | **-** | **-** |
| **GMH5 > Col18A1 (X2)** | **-** | **-** | **-** |

Double transgenic

|  | **Heart rate** | **Failure rate** | **Recovery rate** |
| --- | --- | --- | --- |
| **GMH5 > DSCAM+Col6A2** | ******* | ******* | ******* |
| **GMH5 > DSCAM+Col6A1** | ******* | ******* | ******* |
| **GMH5 > SH3BGR+dCol18A1** | ******* | ******* | ******* |
| **GMH5 > DSCAM+SH3BGR** | **-** | ******* | ******* |
| **GMH5 > DSCAM+dSH3b** | ******* | **-** | ******* |
| **GMH5 > dSH3b+SH3BGR** | ******* | ******* | **-** |
| **GMH5 > Col6A1+Col6A2** | ******* | **-** | **-** |
| **GMH5 > SH3BGR+Col6A1** | ******* | **-** | **-** |
| **GMH5 > dSH3b+Col6A1** | ******* | **-** | **-** |
| **GMH5 > dCol18A1+ Col6A1** | **-** | **-** | **-** |
| **GMH5 > Col6A2+SH3BGR** | **-** | **-** | **-** |
| **GMH5 > dCol18A1+SH3BGR** | **-** | **-** | **-** |
| **GMH5 > dCol18A1+Col6A2** | **-** | **-** | **-** |
