# Supplementary material for: Over-Expression of DSCAM and COL6A2 Cooperatively Generates Congenital Heart Defects
Source: PLoS Genet. 2011 Nov 3;7(11):e1002344. doi: 10.1371/journal.pgen.1002344 (PMC3207880; doi:10.1371/journal.pgen.1002344)
Supplement: Table S3 — Transmission frequencies of DSCAM and COL6A2 transgenes in mice. For double transgenic mice, the frequency of transmission is reduced to 58% of the expected rate (Chi square, P = 1.0904E-07). (DOC) [file pgen.1002344.s007.doc]

**Supporting Table S3:**

|  | **WT** | **DSCAM** | **COL6A2** | **COL6A2/**  **DSCAM** | **Total** |
| --- | --- | --- | --- | --- | --- |
| **Number of animals** | 130 | 139 | 166 | 74 | 509 |
| **Expected %** | 25 | 25 | 25 | 25 | 100 |
| **Actual %** | 25.6 | 27.3 | 32.6 | 14.6 | 100 |
| **P value** |  |  |  | **1.0904E-07** |  |
