# Supplementary material for: Over-Expression of DSCAM and COL6A2 Cooperatively Generates Congenital Heart Defects
Source: PLoS Genet. 2011 Nov 3;7(11):e1002344. doi: 10.1371/journal.pgen.1002344 (PMC3207880; doi:10.1371/journal.pgen.1002344)
Supplement: Table S4 — List of Gene Ontology KEGG pathway genes. (DOC) [file pgen.1002344.s008.doc]

**Supporting Table S**4:

| | Term | Count | % | P-Value | Genes | Pop Hits | Pop Total | Fold Enrichment | | --- | --- | --- | --- | --- | --- | --- | --- | | rno04512:ECM-receptor interaction | 20 | 1.9 | 3.56E-08 | COL4A1,COL3A1,ITGA1,ITGA3,ITGA4,SDC4,COL5A3,COL5A2,COL5A1,HMMR,CD47,ITGA6,ITGB6,COL6A3,COL6A2,COL6A1,LAMC2,TNN,THBS2,SPP1 | 81 | 5590 | 4.540286 | | rno04510:Focal adhesion | 29 | 2.7 | 1.58E-06 | CAV2,CAV1,PDGFB,COL3A1,PXN,MYL9,VCL,ITGB6,COL6A3,SOS2,COL6A2,COL6A1,TNN,SHC2,THBS2,SPP1,COL4A1,PIK3CB,ITGA1,MYLPF,PRKCG,ITGA3,ITGA4,VAV2,COL5A3,COL5A2,COL5A1,ITGA6,LAMC2 | 195 | 5590 | 2.734649 | | rno05410:Hypertrophic cardiomyopathy (HCM) | 15 | 1.4 | 1.41E-04 | CACNA2D1,TGFB3,ITGA1,CACNB1,CACNB2,ITGA3,ITGA4,CACNG1,CACNA2D2,TNNT2,ITGA6,SGCG,ITGB6,PRKAA2,SGCB | 84 | 5590 | 3.2836 | | rno05414:Dilated cardiomyopathy | 15 | 1.4 | 3.00E-04 | CACNA2D1,TGFB3,ITGA1,CACNB1,CACNB2,ITGA3,ITGA4,CACNG1,CACNA2D2,TNNT2,ITGA6,SGCG,PLN,ITGB6,SGCB | 90 | 5590 | 3.064693 | | rno05412:Arrhythmogenic right ventricular cardiomyopathy (ARVC) | 13 | 1.2 | 5.47E-04 | CACNA2D1,ITGA1,CACNB1,CACNB2,ITGA3,ITGA4,CACNG1,CACNA2D2,ITGA6,SGCG,PKP2,ITGB6,SGCB | 74 | 5590 | 3.230352 | | rno04810:Regulation of actin cytoskeleton | 24 | 2.2 | 8.41E-04 | FGFR3,PDGFB,PIK3CB,LIMK1,BAIAP2,FGF11,ITGA1,MYLPF,ITGA3,FGF13,ITGA4,VAV2,IQGAP1,PXN,MYL9,VCL,CHRM3,ITGA6,TIAM1,CHRM1,ITGB6,SOS2,MYH14,CD14 | 209 | 5590 | 2.111559 | | rno05200:Pathways in cancer | 31 | 2.9 | 0.00177 | FGFR3,PDGFB,STAT5A,FGF11,TGFB3,FGF13,WNT4,CASP3,CDKN2B,SOS2,TGFA,FAS,WNT6,MYC,BMP4,BMP2,HSP90AA1,COL4A1,RALBP1,PIK3CB,RXRA,TGFBR2,FZD1,BRCA2,PRKCG,ITGA3,FZD2,RASSF5,ITGA6,LAMC2,TRA1 | 317 | 5590 | 1.798211 | | rno04610:Complement and coagulation cascades | 11 | 1.0 | 0.004122 | A2M,FGG,CD55,MASP1,F3,CD59,SERPINE1,CFH,C1R,C1S,CFD | 70 | 5590 | 2.889568 | | rno04010:MAPK signaling pathway | 26 | 2.4 | 0.004628 | FGFR3,PDGFB,TGFB3,FGF11,CACNB1,CACNB2,FGF13,TNFRSF1A,CASP3,RASGRP3,MAP3K1,SOS2,NFATC4,FAS,MYC,CCDC88B,CACNA2D1,TGFBR2,PRKCG,CACNG1,CACNA2D2,DUSP5,DUSP1,CACNA1G,GADD45A,CD14 | 266 | 5590 | 1.797339 | | rno04360:Axon guidance | 15 | 1.4 | 0.008422 | PLXNA2,LIMK1,EFNB2,EPHB3,EPHA2,SLIT3,EPHA4,SEMA6A,EPHA7,SEMA6C,UNC5B,SEMA3E,SRGAP3,NFATC4,UNC5C | 127 | 5590 | 2.17183 | | rno04670:Leukocyte transendothelial migration | 13 | 1.2 | 0.021172 | PIK3CB,MYLPF,PRKCG,ITGA4,VAV2,PXN,VCL,CLDN15,MYL9,VCAM1,RASSF5,PTK2B,RAPGEF3 | 115 | 5590 | 2.078661 | | rno03320:PPAR signaling pathway | 9 | 0.8 | 0.037036 | CPT1C,LPL,SLC27A1,ACADM,RXRA,SCD,ACSL3,CPT1A,ACOX3 | 71 | 5590 | 2.330893 | | rno04530:Tight junction | 13 | 1.2 | 0.048366 | PARD6B,PARD3,VAPA,MYH2,MYLPF,PRKCG,TJAP1,CLDN15,MYL9,EPB4.1L1,MYH14,PPP2R2B,MYH7B | 130 | 5590 | 1.838816 | | rno04640:Hematopoietic cell lineage | 9 | 0.8 | 0.059172 | CD55,ITGA6,CD3E,CD59,IL4RA,ITGA1,ITGA3,ITGA4,CD14 | 78 | 5590 | 2.121711 | | rno05223:Non-small cell lung cancer | 7 | 0.6 | 0.060477 | RASSF5,PIK3CB,RXRA,SOS2,TGFA,PRKCG,FOXO3 | 52 | 5590 | 2.475329 | | rno04115:p53 signaling pathway | 8 | 0.7 | 0.064783 | STEAP3,CASP3,CCNB2,SERPINE1,FAS,PMAIP1,CCNG2,GADD45A | 66 | 5590 | 2.228868 | | rno04920:Adipocytokine signaling pathway | 8 | 0.7 | 0.069155 | CPT1C,TNFRSF1A,SOCS3,RXRA,PRKAA2,ACSL3,IRS1,CPT1A | 67 | 5590 | 2.195601 | | rno00071:Fatty acid metabolism | 6 | 0.5 | 0.074727 | CPT1C,ACADM,ACSL3,DCI,CPT1A,ACOX3 | 42 | 5590 | 2.62688 | | rno04650:Natural killer cell mediated cytotoxicity | 10 | 0.9 | 0.091204 | CASP3,PTK2B,PIK3CB,SOS2,LOC498276,NFATC4,PRKCG,FAS,VAV2,SHC2 | 100 | 5590 | 1.838816 | | rno04350:TGF-beta signaling pathway | 9 | 0.8 | 0.092978 | BMP4,BMP2,CDKN2B,GDF6,SMAD6,TGFBR2,TGFB3,THBS2,MYC | 86 | 5590 | 1.924342 | | rno05416:Viral myocarditis | 9 | 0.8 | 0.097851 | CAV1,CD55,CASP3,SGCG,MYH2,MYH14,CXADR,MYH7B,SGCB | 87 | 5590 | 1.902223 | |
| --- | --- | --- | --- | --- | --- | --- | --- | --- | --- | --- | --- | --- | --- | --- | --- | --- | --- | --- | --- | --- | --- | --- | --- | --- | --- | --- | --- | --- | --- | --- | --- | --- | --- | --- | --- | --- | --- | --- | --- | --- | --- | --- | --- | --- | --- | --- | --- | --- | --- | --- | --- | --- | --- | --- | --- | --- | --- | --- | --- | --- | --- | --- | --- | --- | --- | --- | --- | --- | --- | --- | --- | --- | --- | --- | --- | --- | --- | --- | --- | --- | --- | --- | --- | --- | --- | --- | --- | --- | --- | --- | --- | --- | --- | --- | --- | --- | --- | --- | --- | --- | --- | --- | --- | --- | --- | --- | --- | --- | --- | --- | --- | --- | --- | --- | --- | --- | --- | --- | --- | --- | --- | --- | --- | --- | --- | --- | --- | --- | --- | --- | --- | --- | --- | --- | --- | --- | --- | --- | --- | --- | --- | --- | --- | --- | --- | --- | --- | --- | --- | --- | --- | --- | --- | --- | --- | --- | --- | --- | --- | --- | --- | --- | --- | --- | --- | --- | --- | --- | --- | --- | --- | --- | --- | --- | --- | --- |
