# Supplementary material for: Over-Expression of DSCAM and COL6A2 Cooperatively Generates Congenital Heart Defects
Source: PLoS Genet. 2011 Nov 3;7(11):e1002344. doi: 10.1371/journal.pgen.1002344 (PMC3207880; doi:10.1371/journal.pgen.1002344)
Supplement: Table S5 — Genes associated with cardiomyopathy. (DOC) [file pgen.1002344.s009.doc]

**Supporting Table S5:** Genes associated with cardiomyopathy.

| **Category** | **-log(pvalue)** | **p-value** | **Molecules** |
| --- | --- | --- | --- |
| Cardiac Hypertrophy | 2.663540266 | 2.17E-03 | INHA,RAB4A,RCAN2,HSPB8,DNAJC3,FOXO3,PNKD (includes EG:25953), CAV1, POSTN, VDR, SERPINE1, AHR, AKAP5, MIF, PLN, NAB1, ANGPT1, RRAD, MYOD1, HBEGF, RAPGEF3, NFATC4, IL33,DUSP1,LAMA4,CXCL12,EDNRA,GATA4 |
| Cardiac Fibrosis | 2.607303047 | 2.47E-03 | ETS1,VAV2,SPP1,PLN,TNNT2,F3,LAMA4,LPL,PNKD (includes EG:25953), POSTN, SERPINE1, ADORA1, AHR |
| Cardiac Arteriopathy | 2.247183569 | 5.66E-03 | SLIT3,DDC,TNNT2,HAPLN3,SMYD3,PXK,DDAH1,PNKD (includes EG:25953),HS6ST3 (includes EG:266722), ADORA2B, SERPINE1, LASS6, TNFRSF9, OLFM2, TKT, HBEGF, CHRNG, SRGAP3, IRS1, FOXN3, SYT1, LRIG1, NCAPD3, DAPK2 (includes EG:23604), BCAS3, CD55, SPATA13, PLXNA2, COL5A1, EFNB2, CD47, CCL2, SNX30, CACNB2, LBP, VCL, NPTXR, ADORA1, UNC5C, NRP2, LARGE, LRRC8D, MMD, GLIPR2, TRIO, PTPRD, SOX6,DOCK8,NFIA,FAM107B,MBNL1,ERMP1,GAS7,LNX1,SERPINA12,SLC38A10,KNTC1,ANK1,SPATA5,SHROOM3,RORA,LPL,POSTN,LPIN2,CAST,LANCL1,TNFRSF11B,EPHA7,CRISPLD2 (includes EG:83716),PTPRE,COL4A1,STK39,GM2A,IL33,SELP,CACNA2D1,PPP1R12B,CD14,PRCP,CHRNB4,CC2D2A,FOXO3,HMGCS1,MTHFD1L,COL5A2,PSRC1,ASB2,SNTB1,GSTO1,CADM1,PNPLA7,LAMA4,TGFA,EDNRA,DSCAM,TOX,PLXDC2 |
| Cardiac Hypoplasia | 2.061980903 | 8.67E-03 | IL6ST,MYCN,RXRA |
| Tachycardia | 1.879426069 | 1.32E-02 | VAV2,TNNT2,ADORA2B,VCL,KCNH2,ADORA1,CHRM3,CHRM1 |
| Cardiac Dilation | 1.53313238 | 2.93E-02 | TGFBR2,GAB2,SPP1,LPL,CAV1,HBEGF,SERPINE1,ADORA1 |
| Cardiac Infarction | 1.53313238 | 2.93E-02 | CD47,SELP,CXCL12,TNNT2,CD55,POSTN,CLU,CAV1,CD14,ADORA2B,ADORA1,COL3A1 |
| Cardiac Proliferation | 1.519993057 | 3.02E-02 | TGFBR2,RXRA,CXADR,GATA4 |
| Cardiac Arrythmia | 1.308034897 | 4.92E-02 | SCN1B,TNNT2,ADORA1,KCNH2,CHRM3,CHRM1 |
| Cardiac Inflammation | 1.283162277 | 5.21E-02 | CLU,CAV1,PNKD (includes EG:25953) |
| Cardiac Enlargement | 1.238072162 | 5.78E-02 | HBEGF,ADORA1 |
| Cardiac Hemorrhaging | 1.238072162 | 5.78E-02 | ITGA4 |
| Cardiac Hyperplasia | 1.238072162 | 5.78E-02 | AHR |
| Cardiac Degeneration | 1.130181792 | 7.41E-02 | LAMA4,CAV1 |
| Heart Failure | 1.130181792 | 7.41E-02 | GAB2,PLN,CTGF,RRAD,TNNT2,CACNA2D1,EDNRA,CACNB2,RAPGEF3,SERPINE1,GATA4,PRKCG |
| Cardiac Stenosis | 1.102372909 | 7.90E-02 | VCAM1,SOX9,SPP1,RUNX2,LIMK1 |
| Cardiac Damage | 0.950781977 | 1.12E-01 | PRKAA2,POSTN,ADORA1,SERPINE1 |
| Cardiac Pulmonary Embolism | 0.950781977 | 1.12E-01 | F2RL2,PTGS1 |
| Bradycardia | 0.673664139 | 2.12E-01 | KCNH1,CHRM3,CHRM1 |
| Cardiac Dysfunction | 0.552841969 | 2.80E-01 | TGFBR2,LPL,CACNB2,SERPINE1 |
| Cardiac Output | 0.347753659 | 4.49E-01 | MLYCD |
| Cardiac Necrosis/Cell Death | 0.334419009 | 4.63E-01 | IL6ST,SOCS3,PTK2B,CASP3,MAP3K1,HSPB8,CACNB2,STAT1,GATA4 |
